# Revealing editing and SNPs of microRNAs in colon tissues by analyzing high-throughput sequencing profiles of small RNAs

**DOI:** 10.1186/1471-2164-15-S9-S11

**Published:** 2014-12-08

**Authors:** Yun Zheng, Ting Li, Ren Ren, Donghua Shi, Shengpeng Wang

**Affiliations:** 1 Faculty of Life Science and Technology, Kunming University of Science and Technology, 727 South Jingming Road, 650500 Kunming, Yunnan, China; 2 School of Life Sciences, Fudan University, 220 Handan Road, 200433 Shanghai, China

**Keywords:** microRNA, RNA editing, SNP, high-throughput sequencing, colon

## Abstract

**Background:**

Editing and mutations in microRNAs (miRNAs) can change the stability of pre-miRNAs and/or complementarities between miRNAs and their targets. Small RNA (sRNA) high-throughput sequencing (HTS) profiles contain miRNAs that are originated from mutated DNAs or are edited during their biogenesis procedures. It is largely unknown whether miRNAs are edited in colon tissues since existing studies mainly focused their attention on the editing of miRNAs in brain tissues.

**Results:**

Through comprehensive analysis of four high-throughput sequencing profiles of normal and cancerous colon tissues, we identified 548 editing and/or SNPs in miRNAs that are significant in at least one of the sequencing profiles used. Our results show that the most abundant editing events of miRNAs in colon tissues are 3'-A and 3'-U. In addition to four known A-to-I editing sites previously reported in brain tissues, four novel A-to-I editing sites are also identified in colon tissues.

**Conclusions:**

This suggests that A-to-I editing of miRNAs potentially is a commonly existing mechanism in different tissues to diversify the possible functional roles of miRNAs, but only a small portion of different miRNAs are edited by the A-to-I mechanism at a significant level. Our results suggest that there are other types of editing in miRNAs through unknown mechanisms. Furthermore, several SNPs in miRNAs are also identified.

## Introduction

MicroRNAs are non-coding RNAs that regulate the expression of protein-coding genes mainly at the post-transcriptional level in plants and animals [1]. MiRNAs have been shown to be edited in multiple ways during their biogenesis [2-14] A widely studied editing is Adenosine-to-Inosine (A-to-I) editing, which is induced by ADAR (adenosine deaminase) on double strand RNAs [12,15]. A-to-I editing converts adenosine residues to inosine residues, which function the same as guanosines [4,11,12]. A-to-I editing can either affect the biogenesis of miRNAs [16-18] or the specificity of miRNA:target complementarity [19]. Another kind of editing happens at the 3' end of mature miRNAs. PAPD4 induced 3' editing of some miRNAs results in adenylation at 3' end of miRNA:miRNA* duplex [7], which potentially interferes the loading of miRNA into RNA-induce silencing complex (RISC). Recently, more evidence showed that the 3' end of some pre-miRNAs are undergoing another type of editing, called mono-uridylation, in which several terminal uridylyl transferases (TUT7, TUT4, TUT2/PAPD4 [13] and TUT1 [20]) introduce an additional U on pre-miRNAs with one nucleotide overhang at 3' end. The mono-uridylation increases the express levels of some miRNAs by facilitating a two nucleotide overhang for Dicer processing [13].

Single Nucleotide Polymorphism (SNP) is a common type of DNA sequence variation throughout the human genome. Similar to editing in miRNAs, SNPs in miRNA genes can affect the function of them by modulating the transcription of the primary transcripts, processing of pri-miRNAs and pre-miRNAs, maturation, or miRNA:target interactions [21,22]. Consequently, the SNPs in miRNAs lead to various diseases, such as chronic lymphocytic leukemia [23], papillary thyroid carcinoma [24], progressive hearing loss [25], and breast cancer [26,27]. Several studies focused on the identification of SNPs in human miRNAs [28-34] have found thousands of SNPs in the pre-miRNAs.

With the fast growth of high throughput sequencing (HTS) technologies, the whole transcriptomes of small RNAs (sRNAs) have become easily available. The huge number of reads from the HTS profiles of sRNAs contain miRNAs that are different from their DNA template, which may caused by either mutations in DNAs or editing on RNAs. Some studies have started to explore the miRNA editing with HTS profiles of sRNAs [5-8,10-13,18] However, the existing methods mainly focused discovery of editing sites, especially A-to-I editing, in brain tissues. The editing of miRNAs in other tissues are still awaiting more researches.

Here, we performed a comprehensive analysis for four sRNA HTS profiles of human colon normal and cancerous samples. Our results show that four editing sites previously identified in brain tissues are also identified in colon tissues. Furthermore, we identified several novel miRNA editing sites, including 4 canonical A-to-I editing sites and several editing sites of other types caused by unknown mechanisms. In addition to these editing of miRNAs, 4 SNPs in miRNAs were also identified in our analysis.

## Materials and methods

### Data sets used

We used four sRNA HTS profiles of cancer and adjacent normal tissues of two stage III colorectal cancer (CRC) patients downloaded from the NCBI Gene Expression Omnibus http://www.ncbi.nlm.nih.gov/geo under series accession number GSE43550 (Shi, Zheng, Ren, Han and Wang, unpublished). One patient underwent surgical resection of CRC tumor directly and the other patient underwent Transcatheter Arterial Infusion chemotherapy (TAI) 1 week before surgical resection. Then, the tumor and adjacent normal samples were used to perform sRNA HTS sequencing using Illumina HiSeq2000 sequencer by following the corresponding protocols. The sequencing profiles of the patient without TAI are named as N_no_TAI (adjacent normal tissue) and T_no_TAI (cancerous tissue), respectively, and the profiles of the patient with TAI are named as N_with_TAI (adjacent normal tissue) and T_with_TAI (cancerous tissue), respectively.

The genome sequences of human were downloaded from UCSC Genome Browser http://genome.ucsc.edu/. The sequences and genomic coordinates of miRNA precursors and mature miRNAs were downloaded from the miRBase release 19 http://www.mirbase.org.

### Preprocessing of small RNA HTS sequencing profiles

The original reads with a low-scored 3' tails were removed firstly. This step is to make sure the clear 3' ends of reads. Then, a self-written program was used to remove the 3' adapters. Then, the unique reads with at least 18 nucleotides and their counts were obtained with self-written programs. The four processed profiles consisted of 15 to 25 million reads, representing 374 to 789 thousand unique sequences.

### Main computational steps

The first step is to align unique reads in sRNA profiles to the pre-miRNAs with NCBI BLASTN. The second step is to obtain the reads mapped to pre-miRNAs. Next, these mapped reads are aligned to the genome with Bowtie [35]. Then, the result of Bowtie is further process to add frequencies of reads and to remove reads with more than 100 aligned loci in the genome. Then, the processed result of Bowtie was analyzed for the possible cross mapping issues of reads with multiple loci in the genome by using the method introduced by de Hoon et al., [6].

Next, we analyzed the mutation and editing (M/E) sites in pre-miRNAs by using several inputs, including the sequences and secondary structure of pre-miRNAs (predicted by RNAfold [36]), the alignments of reads to pre-miRNAs generated by BLASTN, the reads mapped to pre-miRNAs, the alignments of reads against genome generated by Bowtie, and the results of the cross mapping method [6]. In this step, we employed the alignment of BLASTN to obtain the list of read mapped to a pre-miRNA. We then used a modified Smith-Waterman algorithm to align an sRNA read to a pre-miRNA sequence. Briefly, matched and mismatched nucleotides received rewards of +4 and -3, respectively, in alignment. The affine gap penalty, i.e., the penalty increasing linearly with the length of gap after the initial gap opening penalty, was used for gap opening (-4) and gap extension (-2). The weight of reads were retrieved from the results of the cross mapping method [6]. Reads with more than 100 loci were removed before applying the cross mapping method [6]. Thus, we used the initial alignment of reads to the genome generated with Bowtie to examine whether a read had matched loci in genome if it did not appeared in the results of the cross mapping method, or named as missed reads. If a missed read had loci with better alignment scores than its alignment between a pre-miRNA or more than 100 loci of the same alignment scores as its alignment between a pre-miRNA, it was neglected. Otherwise, a missed read was evenly divided to different mapped loci. Then, the results were compared to reported SNPs [34] and editing sites in the DARNED database [37] in miRNAs.

The targets of hsa-miR-6503-3p and edited hsa-miR-6503-3p were predicted with the HitSensor algorithm [38]. Predicted targets with at least 7 continuous Watson-Crick matches in the seed regions were maintained in the analysis.

### *P*-values of identified mutation and editing sites

The quality of identified M/E sites were evaluated using Equation 1 to excluding the probability of being random sequencing errors.

(1) $$Z=\frac{p_{o}-p_{e}}{\sqrt{p_{e}\left(1-p_{e}\right)/n}},$$

Where *p_o _*is the observed percentage of mutated and/or edited reads, *p_e _*is the expected error rate, and *n *is the number of reads matched to the position of pre-miRNA. Since *Z *follows a standard normal distribution, *P*-values of the identified editing or mutation sites can be calculated. *p_e _*is related to the score of sequenced nucleotides. For example, a phred score of 20 will lead to an expected *p_e _*of 1%. Because there could be many mutation and editing sites, the obtained *P*-values were corrected with the Benjamini-Hochberg correction method [39].

### Criteria used to identify significant mutation and editing sites

Chiang et al., [5] proposed three criteria to evaluate the editing sites of miRNAs, i.e., if (i) the relative level of editing is at least 5%; (ii) at least 10 reads support the editing site; and (iii) the editing site is not in the last two positions at the 3' end of mature miRNAs, then the miRNA position was considered to be edited. In addition to the first two criteria of Chiang et al., [5], two more criteria is used in this study, i.e., (i) the score threshold of sequencing reads is 20; and (ii) a multiple-test corrected *P*-value of smaller than 0.05. The third criterion of Chiang et al., [5] was not used here because our aim is to identify all editing and mutation sites, including 3' editing sites, in miRNAs.

### The naming of the editing and mutations in miRNAs

All identified M/E sites are named by the names of the pre-miRNAs, positions of the sites, the nucleotides from the reference pre-miRNA sequences and the edited/mutated nucleotide at the sites. For example, hsa-mir-376a-1_49_A_g is used to mean an A-to-I editing detected at the position 49 of the hsa-mir-376a-1 precursor, the position of the reference sequence is "A" and the edited reads have "g" at this site.

## Results and discussion

### The identified mutation and editing sites

**Figure 1:**
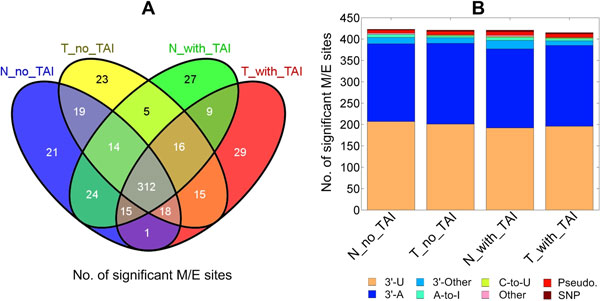
**The number of significant M/E sites in miRNAs and their categories in the analyzed sRNA libraries**.

We totally identified 548 M/E sites of pre-miRNAs that are significant in at least one of the four sequencing profiles used (in additional file 1 Table S1), with around 420 M/E sites in each of the four profiles (see Figure 1). As shown in Figure 1A, 312 M/E sites were commonly identified in all four libraries, suggesting a good repeatability of the identified M/E sites.

Based on the mechanisms of editing or mutation, we classified these significant M/E sites into eight categories as shown in Figure 1B. In addition to editing types with clearly defined mechanisms, i.e., 3'-A, 3'-U, A-to-I and C-to-U, and SNPs, a few other editing sites happened at 3' end of mature miRNAs (classified as 3'-Other in Figure 1B) and other positions of miRNAs (classified as Other) were also identified. We also found that a few editing sites were incorrectly predicted by the reads that were mapped to several loci in the genome. In our analysis, reads with multiple loci might have very small weights, less than 0.05, on loci that were unlikely to generate these reads after applying the cross-mapping correction method [11]. However, the high abundances of these multiple loci reads could still make the editing site become significant because the numbers of the true reads mapped to these loci were relatively small when compared to these incorrectly mapped reads with multiple loci. Thus, these sites were classified as pseudo sites (as Pseudo. in Figure 1B).

### Identification of 3' editing events

The most abundant editing events are 3'-U and 3'-A in all libraries (Figure 1B). In contrast to the reported predominant 3'-U in mature miRNAs from 3' arm of their hairpins [7], our results suggest that a substantial number, 65, of mature miRNAs in the 5' arm also have 5'-U addition (additional file 2 Table S2). Furthermore, 81 mature miRNAs on the 5' arm of their hairpins have 3'-A (additional file 2 Table S2), such as most of the let-7 family members, suggesting that 3' end editing of some miRNAs might happen after the miRNA:miRNA* duplex is formed but before being loaded into the RISC, which is also noticed in literature [7]. Our results of 3'-A on the 5' arm of let-7 family members are also consistent with results in another study [13].

Some miRNAs have 3'-U and 3'-A addition on both the 5' and 3' mature miRNAs (additional file 2 Table S2 and additional file 3 Figure S1). For example, hsa-mir-143 has 3'-U and 3'-A at end of both miR-143-5p and miR-143-3p (see additional file 1 Table S1). The 3' editing on mature miRNAs of the 3' arm of their pre-miRNAs might be added after the pre-miRNA hairpins are formed but before being cut to miRNA:miRNA* duplex by Dicer [7].

Our results show that same miRNAs may have 3'-U and 3'-A at the same positions. For examples, position 33 of hsa-miR-146b, position 48 of hsa-miR-192, and position 37 of hsa-miR-584 locate before the central loop regions and have both 3'-U and 3'-A sites in N_no_TAI and T_no_TAI samples.

In summary, these results suggest that the enzymes that contributes to 3'-A (PAPD4 [7]) and 3'-U (TUT7, TUT4 and PAPD4/TUT2 [13]) may share redundant roles in adding U or A to the end of miRNAs. In fact, the same enzyme may catalyze the addition of both uridine and adenine. As reported previously, PAPD4, also known as TUT2, could introduce both 3'-A [7] and 3'-U editing [13] to miRNAs.

In addition to the 3'-U and 3'-A, our results suggest that some miRNAs also have 3'-G and 3'-C editing. For examples, there are 3'-G addition immediately after hsa-miR-145-3p and hsa-miR-10a-5p (additional file 4 Figure S2A,C and S2B,F). In the mean time, these positions also have 3'-A editing (additional file 4 Figure S2D and G). Further, there are 3'-C at the same position after hsa-miR-145-3p (additional file 4 Figure S2E). Furthermore, hsa-miR-194-2 and hsa-miR-21 also have 3'-C after their 3' mature miRNAs (additional file 5 Figure S3). As reported previously [7,9], 3'-G and 3'-C also happens in some miRNAs in other tissues or cell lines, however their biological relevance is still unclear.

As previously noticed in other tissues, cell lines, and species [7], our results show that the 3'-U and 3'-A addition of miRNAs are also present in colon cancerous and adjacent normal tissues.

### A-to-I editing of miRNAs in colon tissues

As shown in Table 1, we totally identified 14 editing sites in pre-miRNAs that satisfy a combination of criteria, i.e., at least ten edited reads, an edited reads percentage of at least 5%, and a multiple-test corrected *P*-value of less than 0.05, in at least one of the four sequencing profiles used.

**Table 1:** The identified editing sites in miRNAs in the sequenced stage III CRC samples.

					N_no_TAI			T_no_TAI			N_with_TAI			T_with_TAI			
**ME ID**	**P.P.**	**S.P**.	**W**	**ME**	**MER**	**MEP**	**FDR_P**	**MER**	**MEP**	**FDR_P**	**MER**	**MEP**	**FDR_P**	**MER**	**MEP**	**FDR_P**	**Ref**.

hsa-mir-376a-1_49_A _g	49	6	A	g	23	89.4	0.00E+00	19	88.7	0.00E+00	28	91.7	0.00E+00	55	92.3	0.00E+00	[3,4]
hsa-mir-376a-2_55_A_g	55	6	A	g	23	89.4	0.00E+00	19	88.7	0.00E+00	28	91.7	0.00E+00	55	92.3	0.00E+00	[4]
hsa-mir-376c_48_A_g	48	6	A	g	35	35.2	0.00E+00	26	31	0.00E+00	35	38.4	0.00E+00	70	34.5	0.00E+00	[4]
hsa-let-7a-2_28_A_g	28		A	g	33	17.1	1.90E-13	41	23.6	0.00E+00	63	29	0.00E+00	34	25.5	0.00E+00	
hsa-mir-378b_53_A_g	53		A	g	24	92.4	0.00E+00	6	100	0.00E+00	26	95.5	0.00E+00	15	92.3	0.00E+00	
hsa-mir-497_83_A_g	83		A	g	15	83.3	0.00E+00	3	72.7	3.71E-10	19	85.7	0.00E+00	6	83.3	0.00E+00	
hsa-mir-6503_59_A_g	59	7	A	g	15	72.7	0.00E+00	6	46.9	3.11E-11	9	62.5	0.00E+00	6	45.5	2.03E-11	
hsa-mir-411_20_A_g	20	5	A	g	36	4.5	1.00E+00	51	4.8	1.00E+00	43	9.7	4.84E-05	38	5	1.00E+00	[4]
hsa-mir-125b-1_25_C_u	25		C	u	226	5.1	1.00E+00	197	6.9	4.28E-05	392	8.9	0.00E+00	191	6.7	3.00E-04	
hsa-mir-125b-2_27_C_u	27		C	u	225	5	1.00E+00	195	6.8	7.50E-05	392	8.8	0.00E+00	192	6.6	4.69E-04	
hsa-mir-141_72_U_g	72		U	g	747	5.6	8.11E-03	1034	4.4	1.00E+00	482	4.7	1.00E+00	2067	4.1	1.00E+00	
hsa-mir-378c_30_-_g	30		-	g	57	21.8	0.00E+00	21	22.7	5.90E-14	55	27.8	0.00E+00	17	32.5	0.00E+00	
hsa-mir-429_64_U_g	64		U	g	239	6.5	2.05E-04	261	5.4	1.00E+00	149	6.6	2.83E-03	155	5.5	7.63E-01	
hsa-mir-375_56_G_c	56		G	c	249	1.9	1.00E+00	163	1.2	1.00E+00	610	5.6	2.18E-02	17	1.6	1.00E+00	

P.P. and S.P. means the position in pre-miRNA and seed region, respectively. W and ME means the original nucleotide, as in the miRBase, and edited/mutated nucleotide, respectively. N_no_TAI, T_no_TAI, N_with_TAI (adjacent normal tissue) and T_with_TAI means the sequencing profiles of adjacent normal tissue without TAI, cancer tissue without TAI, adjacent normal tissue with TAI and cancerous tissue with TAI, respectively. MER, MEP, and FDR_P means the number of edited reads, the percentage of edited reads, and the FDR-corrected *P*-value using method of [39], respectively.

8 out of the 14 editing sites are A-to-I editing, representing the largest category. As shown in Table 1, four of these 8 A-to-I sites had been reported previously in brain tissues [3,4].

**Figure 2:**
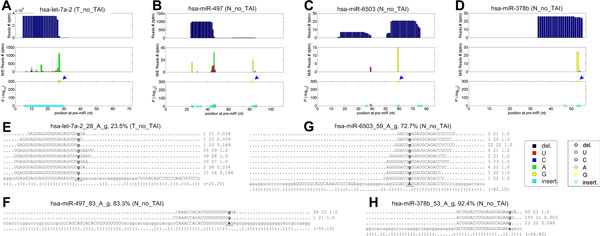
**The details of four identified A-to-I editing sites in miRNAs**. (A) to (D) are schematic views of hsa-miR-378b, hsa-miR-497, hsa-miR-6503 and hsa-let-7a-2, respectively. In part (A) to (D), the upper panel shows the total number of reads (vertical axis) aligned to positions (horizontal axis) of the precursors of miRNAs, the central panel shows the number of reads that do not match the reference precursor sequences of miRNAs, the lower panel shows *- *log10 of the multiple-test corrected *P*-value (with Benjamini and Hochberg [39] method) of the editing or mutation sites. The meaning of different colors in the central and lower panels are given at the lower right corner. (E) to (H) are the reads supporting hsa-miR-378b_53_A_g, hsa-miR-497_83_A_g, hsa-miR-6503_59_A_g and hsa-let-7a-a_28_A_g, respectively. In part (E) to (H), the lower case nucleotides in the reads mean the mismatched nucleotides that are generated in editing or mutation events; and the upper case nucleotides in the precursors mean the mature miRNAs. The three columns after a read are the original number of this read in the library, the length of the read, and the weight of this read at this locus calculated by the cross mapping algorithm [11]. The percentage values after the name of editing site are the percentage of edited or mutated reads. The lines below the pre-miRNA sequences are the secondary structures predicted with RNAfold [36], and numbers in the parent thesis mean the minimum free energy in k-cal/mol. In all parts, the names of the sequencing profiles are shown in the parenthesis after the name of the editing sites.

Four newly identified A-to-I editing sites are shown in Figure 2. hsa-let-7a-a_28_A_g in Figure 2A is close to 3' end of mature let-7a-2. But as shown in Figure 2E, 84 reads with 26 to 29 nucleotides at this locus clearly demonstrate that this is unlikely to be an 3' addition site since there are as many as 5 nucleotides after this A-to-I site. As shown in Figure 2B to 2D, these three editing sites all happen in the regions of mature miRNAs. As reported previously, UAG is a preferred motif for A-to-I editing [3,4,11]. The newly identified A-to-I editing sites also have a preference for UAG motif. Three newly identified sites have a local motif of UAG, as shown in Figure 2E to 2G, and hsa-miR-378b_53_A_g has a local motif of AAG, which is also frequently modified as noticed previously [4].

Existing studies of miRNA editing mainly focused their attention on the A-to-I sites in brain tissues of mammals [5,11,12]. Here our results show that there are some editing sites, including a few A-to-I sites, in colon cancer and corresponding adjacent normal tissues. This suggests that miRNA editing is potentially a widely used mechanism to realize more diverse roles of miRNAs in different tissues and organs. However, only a limited number of miRNAs are edited by the A-to-I mechanism which is similar to previous results in brain [11].

Our results indicate that four known A-to-I editing sites on hsa-miR-376a-1, hsamiR-376a-2, hsa-miR-376c and hsa-miR-411 are also edited in normal and/or cancerous colon tissues, although existing studies mainly reported these sites in brain tissues [3,4,11].

### Other types of miRNA editing in colon tissues

**Figure 3:**
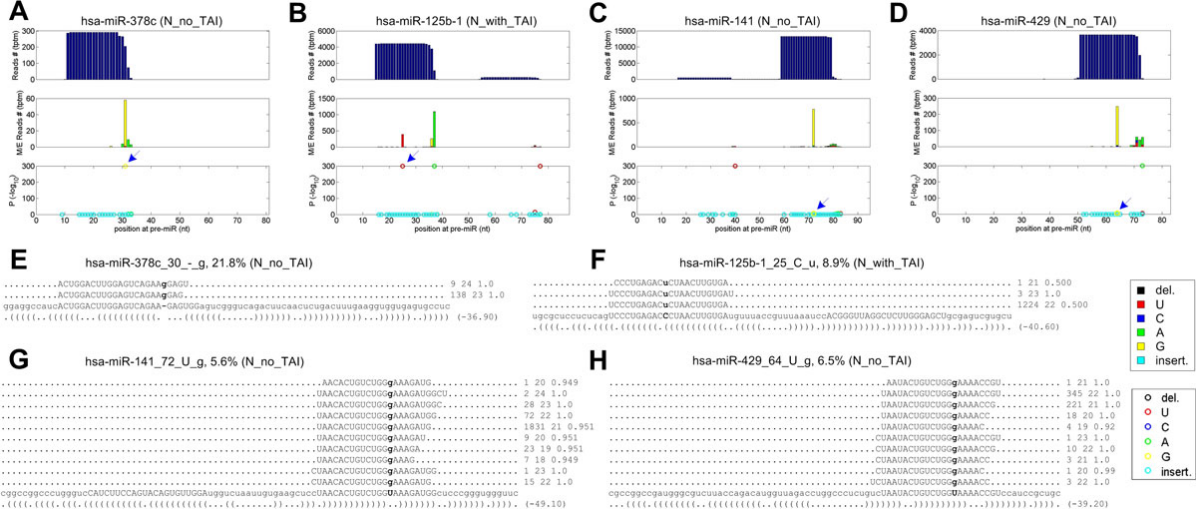
**The details of four non-A-to-I identified editing sites in miRNAs**. (A) to (D) are the schematic views of hsa-miR-3653, hsa-miR-194-2, hsa-miR-378c and hsa-miR-21, respectively. (E) to (H) are the reads supporting hsa-miR-3653_42_C_u, hsa-miR-194-2_37_A_c, hsa-miR-378c_30_-_g, and hsa-miR-21_32_G_c, respectively. Legend idem to those of Figure 2.

As shown in Table 1, we detected six non-canonical A-to-I editing sites in our data sets. Four of them are shown in Figure 3 hsa-miR-378c_30_-_g in Figure 3A is an insertion of "g" to the mature hsa-miR-378c. This editing contributes to 21.8% to 32.5% of the sequenced reads of hsa-miR-378c and is significant in all four sequencing profiles used (Table 1), suggesting that a few miRNAs may have insertions after being transcribed. However, the mechanism of this editing still needs further researches.

C-to-U editing is detected in hsa-miR-125b-1 and hsa-miR-125b-2 in all four sequencing libraries analyzed (Table 1 and Figure 3B). Previous studies also found several miRNAs, such as miR-379, miR-140*, and miR376a, may have C-to-U editing, probably occurring only in preor mature miRNAs [12].

Two U-to-G editing are detected at hsa-miR-141 and hsa-miR-429 (Figure 3C and 3D). And hsa-miR-375 has an G-to-C editing at position 56 of its precursor (Table 1). Similar to the insertion at hsa-miR-378c, the mechanisms of these several editing sites are still unclear and need more studies. Another possible reason for the three non-canonical A-to-I editing in Figure 3F, G and 3H are SNPs. However the low percentages of M/E reads from these loci suggest that these sites are unlikely to be inherited SNPs, and at most they could be somatic SNPs.

### Identification of SNPs in miRNAs in the samples used

**Figure 4:**
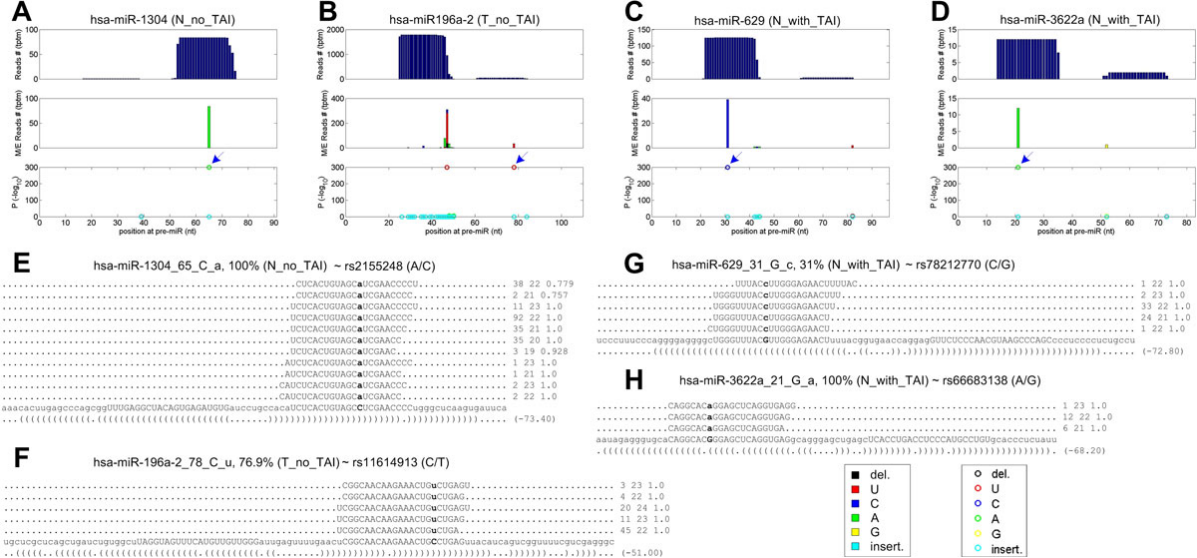
**The details of four identified SNPs in miRNAs**. (A) to (D) are the schematic views of hsa-miR-1304, hsa-miR-196a-2, hsa-miR-629 and hsa-miR-3676, respectively. (E) to (H) are the reads supporting hsa-miR-1304_65_C_a, hsa-miR-196a-2_78_C_u, hsa-miR-629_31_G_c and hsa-miR-3662a_21_G_a, respectively. Legend idem to those of Figure 2. In part (E) to (H), the corresponding rs ID of the SNPs are given after the ID of the M/E site.

We identifies four significant SNP sites as shown in Figure 4. hsa-miR-1304_65_C_a, corresponding to SNP rs2155248, universally appears in all four samples with 100% mutated/edited reads percentage (Figure 4A and 4E).

hsa-miR-196a-2_78_C_u, corresponding to an SNP rs11614913, is only significant in the T_no_TAI sample (Figure 4B and 4F). 76.9% reads covering this SNP are mutated in this sample (Figure 4F). Previous study showed that rs11614913 in hsa-miR-196a-2 reduced the expression level of miR-196a, and was significantly associated with decreased breast cancer risk [26]. Other researches also reported that the high levels of miR-196a may promote oncogenic phenotype of colorectal cancer cells [40] and non-small cell lung cancer cell [41]. The appearance of mutated hsa-miR-196a-2 in the T_no_TAI sample does not agree with the oncogenic roles of hsa-miR-196a-2, suggesting that the tumor genesis of this patient may caused by other biological pathways.

hsa-miR-629 31 G c is caused by an SNP rs78212770 (Figure 4C and 4G) and does not shown in two samples N_no_TAI and T_no_TAI. 31.0% and 21.1% reads in the N_with_TAI and T_with_TAI samples from this locus are mutated (see Figure 4G), suggesting it is a somatic mutation in this patient.

hsa-miR-3662a_62_G_a caused by an SNP rs66683138 appears in 100% reads from this locus in the N_with_TAI sample (Figure 4D and 4H). Other samples also have a few reads that support the existence of this SNP in those samples.

In addition to the four SNPs in Figure 4, our results also suggest that hsa-miR-3676_62_G_a is a potential SNP since it happens with 100% reads from this locus in all four libraries (see additional file 1 Table S1 and additional file 6 Figure S4). hsa-miR-3676 is annotated as a tRNA since release 20 of the miRBase. Thus, this result indicates that small RNA sequencing profiles potentially could be used to find SNPs in tRNAs as well.

### The targets of edited hsa-miR-6503-3p

**Figure 5:**
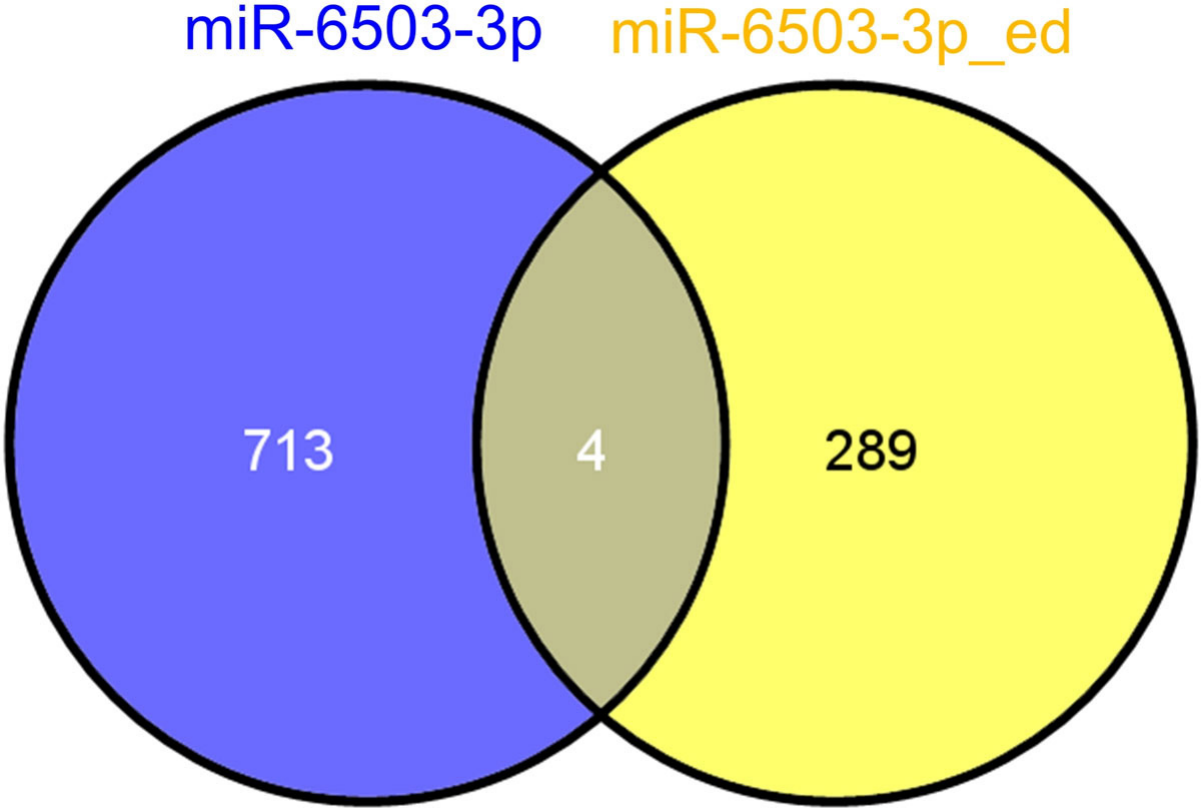
**The number of targets of hsa-miR-6503-3p (blue circle) and edited hsa-miR-6503-3p (yellow circle)**.

As shown in Table 1 and Figure 2C and 2G, there is an A-to-G editing site in the seed region of hsa-miR-6503-3p which will potentially change the complementarities between this miRNA and its targets. Thus, we predicted the potential targets for both original hsa-miR-6503-3p and edited hsa-miR-6503-3p. As shown in Figure 5, hsa-miR-6503-3p has 717 targets but the edited hsa-miR-6503-3p only has 293 targets, and original and edited hsa-miR-6503-3p only shared 4 targets. These results suggest that the A-to-I editing at position 7 of the seed of hsa-miR-6503-3p severely changes the complementarities between this miRNA and its targets, and changes the function of hsa-miR-6503-3p.

## Conclusion

Our results suggest that some miRNAs are edited in colon tissues after analyzing four colon normal or cancerous small RNA HTS sequencing profiles. 3'-A and 3'-U are the two most common editing events in the examined tissues. Four novel A-to-I editing sites on miRNAs, as well as another four A-to-I editing sites previously reported in brain tissues, are characterized in colon normal and/or cancerous tissues. Several editing sites of other types are also identified, however their mechanisms need more researches. Our results identify four SNPs that universally appeared with M/E read percentages of 100% in all of the four samples used or with less than 100% M/E reads percentages in some of the samples used, suggesting inherited or somatic mutations respectively.

## Competing interests

The authors declare that they have no competing interests.

## Authors' contributions

YZ conceived of, designed and coordinated this research; implemented the computational pipeline; and wrote the manuscript. YZ, TL and RR performed the computational experiments. YZ, TL, RR and SW analyzed the results. RR and DS collected the RNA samples.

## Supplementary Material

Additional File 1 **Table S1 -- The mutation and editing sites of miRNAs identified in colon tissues**. The meanings of columns are given in the second sheet. Click here for file

Additional File 2 Table S2 -- miRNAs with 3'-A and 3'-U sites that are significant in all of the four libraries used. The miRNA with * and # means sites that were also reported previously in four and three cell lines, respectively, by Burroughs et al., [7]. Click here for file

Additional File 3 **Figure S1 -- The number of pre-miRNAs with 3'-A and 3'-U sites in colon tissues**. 3'-A, 3'-A CL, 3'-U and 3'-U CL means the number of pre-miRNAs that have 3'-A editing on the mature miRNA of 3' arm, 3'-A editing on the mature miRNA of 5' arm, 3'-U editing on the mature miRNA of 3' arm, and 3'-U on the 5' arm of their hairpin structures. Click here for file

Additional File 4 **Figure S2 -- The examples of 3'-G editing of miRNAs**. (A) and (B) are schematic views of hsa-miR-145 and hsa-miR-10a, respectively. (C) to (G) are the reads supporting hsa-miR-145_76_U_g, hsa-miR-145_76_U_a, hsa-miR-145_76_U_c, hsa-miR-10a_45_U_g, hsa-miR-10a_45_U_a, respectively. Legend idem to those of Figure 2. Click here for file

Additional File 5 **Figure S3 -- The examples of 3'-C editing of miRNAs**. (A) and (B) are schematic views of hsa-miR-194-2 and hsa-miR-21, respectively. (C) to (D) are the reads supporting hsa-miR-194-2_37_A_c and hsa-miR-21_32_G_c, respectively. Legend idem to those of Figure 2. Click here for file

Additional File 6 **Figure S4 -- The example of a potential SNP on hsa-miR-3676**. (A) is a schematic view of hsa-miR-3676. (B) to (C) are the reads supporting hsa-miR-3676_62_G_a in the N_no_TAI and T_no_TAI data set, respectively. Legend idem to those of Figure 2. Click here for file

## Declarations

Publication charges for this work was funded by a start-up grant (No. 14078285) of Kunming University of Science and Technology to YZ.

This article has been published as part of *BMC Genomics *Volume 15 Supplement 9, 2014: Thirteenth International Conference on Bioinformatics (InCoB2014): Computational Biology. The full contents of the supplement are available online at http://www.biomedcentral.com/bmcgenomics/supplements/15/S9.

## References

[B1] Bartel DP MicroRNAs: Genomics, biogenesis, mechanism, and function Cell 2004 116 281 297 10.1016/S0092-8674(04)00045-5 14744438

[B2] Luciano DJ Mirsky H Vendetti NJ Maas S RNA editing of a miRNA precursor RNA 2004 10 8 1174 1177 10.1261/rna.7350304 15272117 PMC1370607

[B3] Blow MJ Grocock RJ Van Dongen S Enright AJ Dicks E Futreal PA Wooster R Stratton MR RNA editing of human microRNAs Genome Biology 2006 7 4 27 10.1186/gb-2006-7-4-r27 PMC1557993 16594986

[B4] Kawahara Y Megraw M Kreider E Iizasa H Valente L Hatzigeorgiou AG Nishikura K Frequency and fate of microRNA editing in human brain Nucleic Acids Research 2008 36 16 5270 5280 10.1093/nar/gkn479 18684997 PMC2532740

[B5] Chiang HR Schoenfeld LW Ruby JG Auyeung VC Spies N Baek D Johnston WK Russ C Luo S Babiarz JE Blelloch R Schroth GP Nusbaum C Bartel DP Mammalian microRNAs: experimental evaluation of novel and previously annotated genes Genes & Development 2010 24 10 992 1009 10.1101/gad.1884710 20413612 PMC2867214

[B6] de Hoon MJL Taft RJ Hashimoto T Kanamori-Katayama M Kawaji H Kawano M Kishima M Lassmann T Faulkner GJ Mattick JS Daub CO Carninci P Kawai J Suzuki H Hayashizaki Y Cross-mapping and the identification of editing sites in mature microRNAs in high-throughput sequencing libraries Genome Research 2010 20 2 257 264 10.1101/gr.095273.109 20051556 PMC2813481

[B7] Burroughs AM Ando Y de Hoon MJL Tomaru Y Nishibu T Ukekawa R Funakoshi T Kurokawa T Suzuki H Hayashizaki Y Daub CO A comprehensive survey of 3' animal miRNA modification events and a possible role for 3' adenylation in modulating miRNA targeting effectiveness Genome Research 2010 20 10 1398 1410 10.1101/gr.106054.110 20719920 PMC2945189

[B8] Guo L Yang Q Lu J Li H Ge Q Gu W Bai Y Lu Z A comprehensive survey of miRNA repertoire and 3' addition events in the placentas of patients with pre-eclampsia from high-throughput sequencing PloS ONE 2011 6 6 21072 10.1371/journal.pone.0021072 PMC3120834 21731650

[B9] Wyman SK Knouf EC Parkin RK Fritz BR Lin DW Dennis LM Krouse MA Webster PJ Tewari M Post-transcriptional generation of miRNA variants by multiple nucleotidyl transferases contributes to miRNA transcriptome complexity Genome Research 2011 21 9 1450 1461 10.1101/gr.118059.110 21813625 PMC3166830

[B10] Mizuguchi Y Mishima T Yokomuro S Arima Y Kawahigashi Y Shigehara K Kanda T Yoshida H Uchida E Tajiri T Sequencing and bioinformatics-based analyses of the microRNA transcriptome in Hepatitis B-related hepatocellular carcinoma PloS ONE 2011 6 1 15304 10.1371/journal.pone.0015304 PMC3026781 21283620

[B11] Alon S Mor E Vigneault F Church GM Locatelli F Galeano F Gallo A Shomron N Eisenberg E Systematic identification of edited microRNAs in the human brain Genome Research 2012 22 8 1533 1540 10.1101/gr.131573.111 22499667 PMC3409266

[B12] Ekdahl Y Farahani HS Behm M Lagergren J Öhman M A-to-I editing of microRNAs in the mammalian brain increases during development Genome Research 2012 22 8 1477 1487 10.1101/gr.131912.111 22645261 PMC3409261

[B13] Heo I Ha M Lim J Yoon MJJ Park JEE Kwon SC Chang H Kim VN Mono-Uridylation of Pre-MicroRNA as a key step in the biogenesis of group II let-7 MicroRNAs Cell 2012 151 3 521 532 10.1016/j.cell.2012.09.022 23063654

[B14] García-López J Hourcade JdD del Mazo J Reprogramming of microRNAs by adenosine-to-inosine editing and the selective elimination of edited microRNA precursors in mouse oocytes and preimplantation embryos Nucleic Acids Research 2013 41 10 5483 5493 10.1093/nar/gkt247 23571754 PMC3664825

[B15] Bass B Nishikura K Keller W Seeburg PH Emeson R O'connell M Samuel C Herbert A A standardized nomenclature for adenosine deaminases that act on RNA RNA 1997 3 9 947 9292492 PMC1369539

[B16] Yang W Chendrimada TP Wang Q Higuchi M Seeburg PH Shiekhattar R Nishikura K Modulation of microRNA processing and expression through RNA editing by ADAR deaminases Nature Structural & Molecular Biology 2005 13 1 13 21 16369484 10.1038/nsmb1041 PMC2950615

[B17] Kawahara Y Zinshteyn B Chendrimada TP Shiekhattar R Nishikura K RNA editing of the microRNA-151 precursor blocks cleavage by the Dicer-TRBP complex EMBO Reports 2007 8 8 763 769 10.1038/sj.embor.7401011 17599088 PMC1978079

[B18] Vesely C Tauber S Sedlazeck FJ von Haeseler A Jantsch MF Adenosine deaminases that act on RNA induce reproducible changes in abundance and sequence of embryonic miRNAs Genome Research 2012 22 8 1468 1476 10.1101/gr.133025.111 22310477 PMC3409260

[B19] Kawahara Y Zinshteyn B Sethupathy P Iizasa H Hatzigeorgiou AG Nishikura K Redirection of silencing targets by Adenosine-to-Inosine editing of miRNAs Science 2007 315 5815 1137 1140 10.1126/science.1138050 17322061 PMC2953418

[B20] Knouf EC Wyman SK Tewari M The human TUT1 nucleotidyl transferase as a global regulator of microRNA abundance PloS ONE 2013 8 7 69630 10.1371/journal.pone.0069630 PMC3715485 23874977

[B21] Duan R Pak CH Jin P Single nucleotide polymorphism associated with mature miR-125a alters the processing of pri-miRNA Human Molecular Genetics 2007 16 9 1124 1131 10.1093/hmg/ddm062 17400653

[B22] Ryan BM Robles AI Harris CC Genetic variation in microRNA networks: the implications for cancer research Nature Reviews Cancer 2010 10 6 389 402 10.1038/nrc2867 20495573 PMC2950312

[B23] Calin GA Ferracin M Cimmino A Di Leva G Shimizu M Wojcik SE Iorio MV Visone R Sever NI Fabbri M A MicroRNA signature associated with prognosis and progression in chronic lymphocytic leukemia New England Journal of Medicine 2005 353 17 1793 1801 10.1056/NEJMoa050995 16251535

[B24] Jazdzewski K Murray EL Franssila K Jarzab B Schoenberg DR de La Chapelle A Common SNP in pre-miR-146a decreases mature miR expression and predisposes to papillary thyroid carcinoma Proceedings of the National Academy of Sciences of the United States of America 2008 105 20 7269 7274 10.1073/pnas.0802682105 18474871 PMC2438239

[B25] Á Mencía Modamio-Høybjør S Redshaw N Morín M Mayo-Merino F Olavarrieta L Aguirre LA Del Castillo I Steel KP Dalmay T Mutations in the seed region of human miR-96 are responsible for nonsyndromic progressive hearing loss Nature Genetics 2009 41 5 609 613 10.1038/ng.355 19363479

[B26] Hoffman AE Zheng T Yi C Leaderer D Weidhaas J Slack F Zhang Y Paranjape T Zhu Y microRNA miR-196a-2 and breast cancer: a genetic and epigenetic association study and functional analysis Cancer Research 2009 69 14 5970 5977 10.1158/0008-5472.CAN-09-0236 19567675 PMC2716085

[B27] Gao LB Bai P Pan XM Jia J Li LJ Liang WB Tang M Zhang LS Wei YG Zhang L The association between two polymorphisms in pre-miRNAs and breast cancer risk: a meta-analysis Breast Cancer Research and Treatment 2011 125 2 571 574 10.1007/s10549-010-0993-x 20640596

[B28] Bhartiya D Laddha SV Mukhopadhyay A Scaria V miRvar: A comprehensive database for genomic variations in microRNAs Human Mutation 2011 32 6 2226 2245 10.1002/humu.21482 21618345

[B29] Saunders MA Liang H Li WH Human polymorphism at microRNAs and microRNA target sites Proceedings of the National Academy of Sciences of the United States of America 2007 104 9 3300 3305 10.1073/pnas.0611347104 17360642 PMC1805605

[B30] Iwai N Naraba H Polymorphisms in human pre-miRNAs Biochemical and Biophysical Research Communications 2005 331 4 1439 1444 10.1016/j.bbrc.2005.04.051 15883035

[B31] Gong J Tong Y Zhang HM Wang K Hu T Shan G Sun J Guo AY Genome-wide identification of SNPs in microRNA genes and the SNP effects on microRNA target binding and biogenesis Human Mutation 2012 33 1 254 263 10.1002/humu.21641 22045659

[B32] Lu J Clark AG Impact of microRNA regulation on variation in human gene expression Genome Research 2012 22 7 1243 1254 10.1101/gr.132514.111 22456605 PMC3396366

[B33] Zorc M Skok DJ Godnic I Calin GA Horvat S Jiang Z Dovc P Kunej T Catalog of MicroRNA Seed Polymorphisms in Vertebrates PloS ONE 2012 7 1 30737 10.1371/journal.pone.0030737 PMC3267754 22303453

[B34] Han M Zheng Y Comprehensive analysis of single nucleotide polymorphisms in human MicroRNAs PLoS ONE 2013 8 11 78028 10.1371/journal.pone.0078028 PMC3818353 24223755

[B35] Langmead B Trapnell C Pop M Salzberg S Ultrafast and memory-efficient alignment of short DNA sequences to the human genome Genome Biology 2009 10 3 25 10 10.1186/gb-2009-10-3-r25 PMC2690996 19261174

[B36] Hofacker IL Vienna RNA secondary structure server Nucleic Acids Research 2003 31 13 3429 3431 10.1093/nar/gkg599 12824340 PMC169005

[B37] Kiran A Baranov PV DARNED: a DAtabase of RNa EDiting in humans Bioinformatics 2010 26 14 1772 1776 10.1093/bioinformatics/btq285 20547637

[B38] Zheng Y Zhang W Animal microRNA target prediction using diverse sequence-specific determinants Journal of Bioinformatics and Computational Biology 2010 8 4 763 788 10.1142/S0219720010004896

[B39] Benjamini Y Hochberg Y Controlling the false discovery rate: a practical and powerful approach to multiple testing Journal of the Royal Statistical Society Series B (Methodological) 1995 57 1 289 300

[B40] Schimanski CC Frerichs K Rahman F Berger M Lang H Galle PR Moehler M Gockel I High miR-196a levels promote the oncogenic phenotype of colorectal cancer cells World journal of gastroenterology: WJG 2009 15 17 2089 10.3748/wjg.15.2089 19418581 PMC2678579

[B41] Liu Xh Lu Kh Wang Km Sun M Zhang Eb Yang Js Yin Dd Liu Zl Zhou J Liu Zj MicroRNA-196a promotes non-small cell lung cancer cell proliferation and invasion through targeting HOXA5 BMC Cancer 2012 12 1 348 10.1186/1471-2407-12-348 22876840 PMC3503718

