# Supplementary figures and images for: Revealing editing and SNPs of microRNAs in colon tissues by analyzing high-throughput sequencing profiles of small RNAs
Source: BMC Genomics. 2014 Dec 8;15(Suppl 9):S11. doi: 10.1186/1471-2164-15-S9-S11 (PMC4290591; doi:10.1186/1471-2164-15-S9-S11)

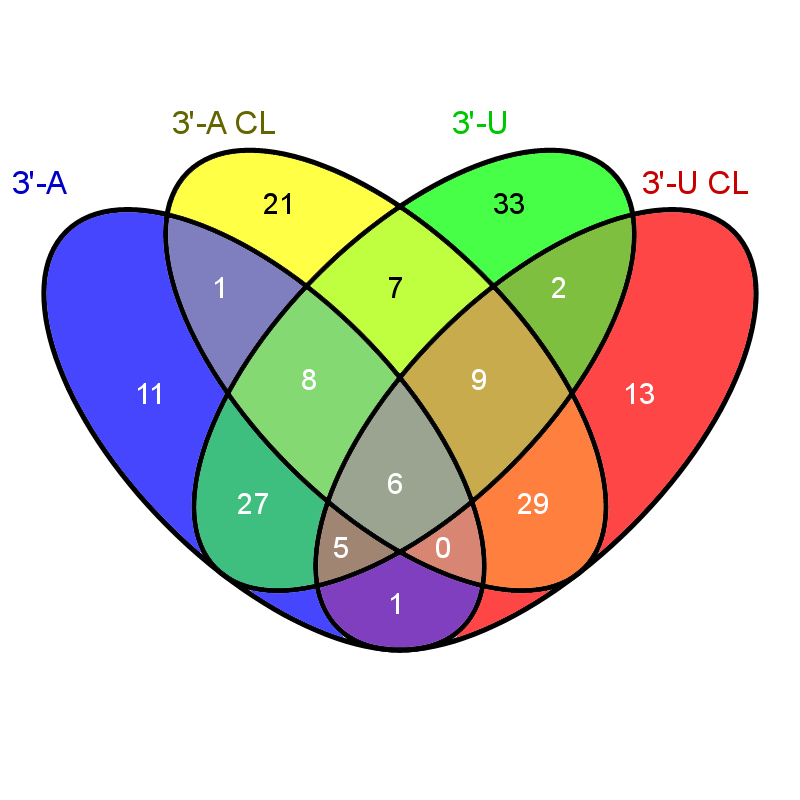

Supplement: Additional File 3 — Figure S1 -- The number of pre-miRNAs with 3'-A and 3'-U sites in colon tissues. 3'-A, 3'-A CL, 3'-U and 3'-U CL means the number of pre-miRNAs that have 3'-A editing on the mature miRNA of 3' arm, 3'-A editing on the mature miRNA of 5' arm, 3'-U editing on the mature miRNA of 3' arm, and 3'-U on the 5' arm of their hairpin structures. [file 1471-2164-15-S9-S11-S3.png]

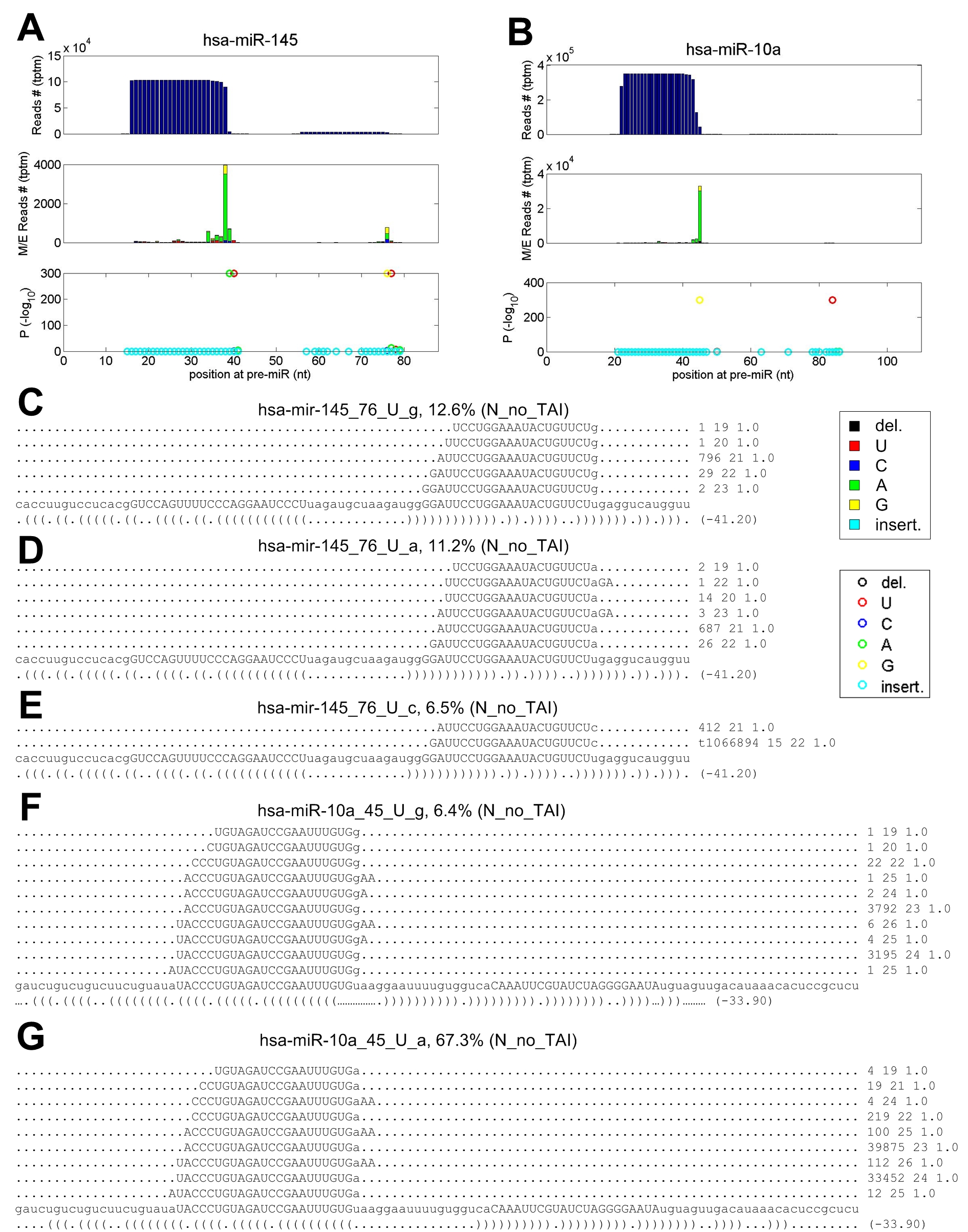

Supplement: Additional File 4 — Figure S2 -- The examples of 3'-G editing of miRNAs. (A) and (B) are schematic views of hsa-miR-145 and hsa-miR-10a, respectively. (C) to (G) are the reads supporting hsa-miR-145_76_U_g, hsa-miR-145_76_U_a, hsa-miR-145_76_U_c, hsa-miR-10a_45_U_g, hsa-miR-10a_45_U_a, respectively. Legend idem to those of Figure 2. [file 1471-2164-15-S9-S11-S4.jpg]

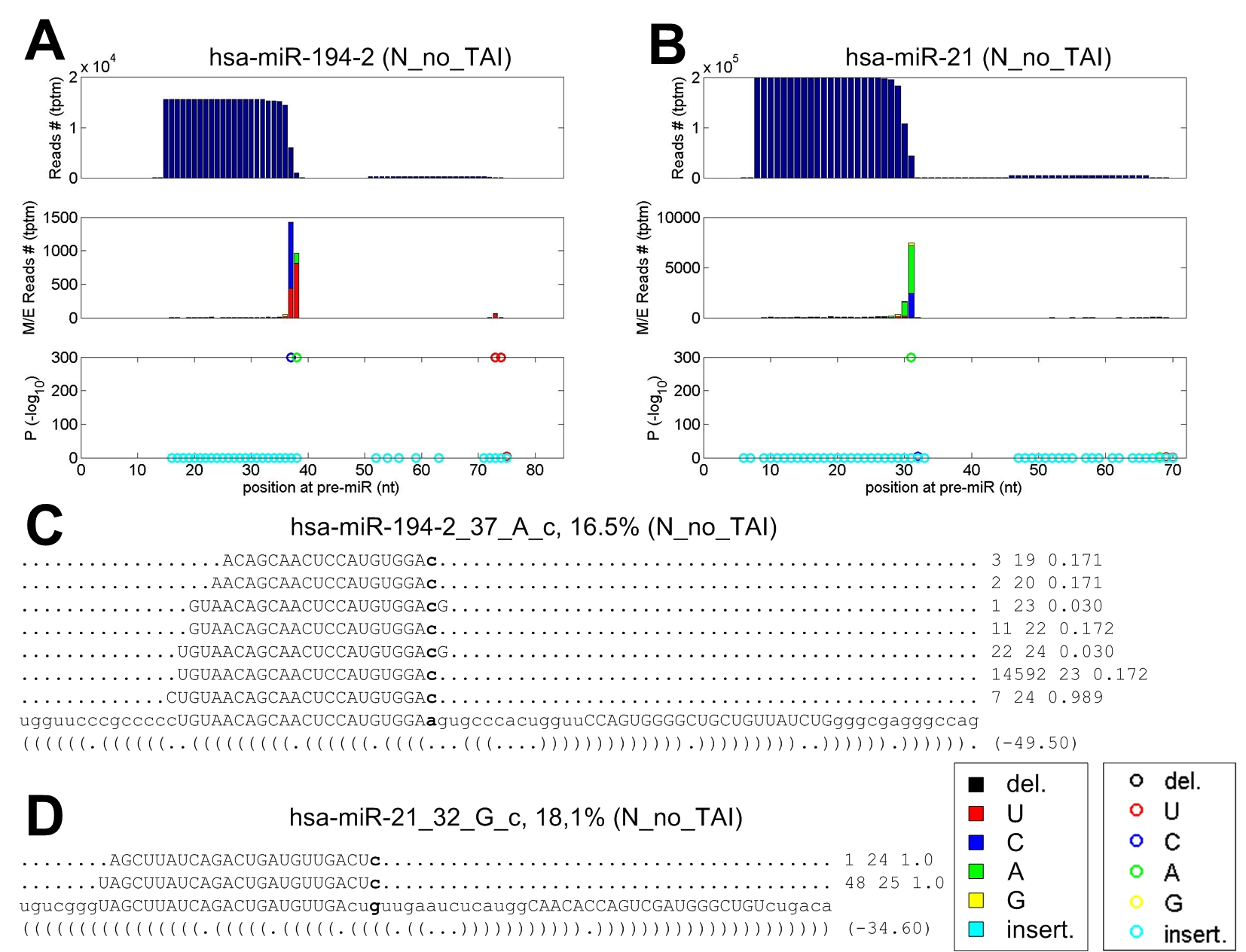

Supplement: Additional File 5 — Figure S3 -- The examples of 3'-C editing of miRNAs. (A) and (B) are schematic views of hsa-miR-194-2 and hsa-miR-21, respectively. (C) to (D) are the reads supporting hsa-miR-194-2_37_A_c and hsa-miR-21_32_G_c, respectively. Legend idem to those of Figure 2. [file 1471-2164-15-S9-S11-S5.jpg]

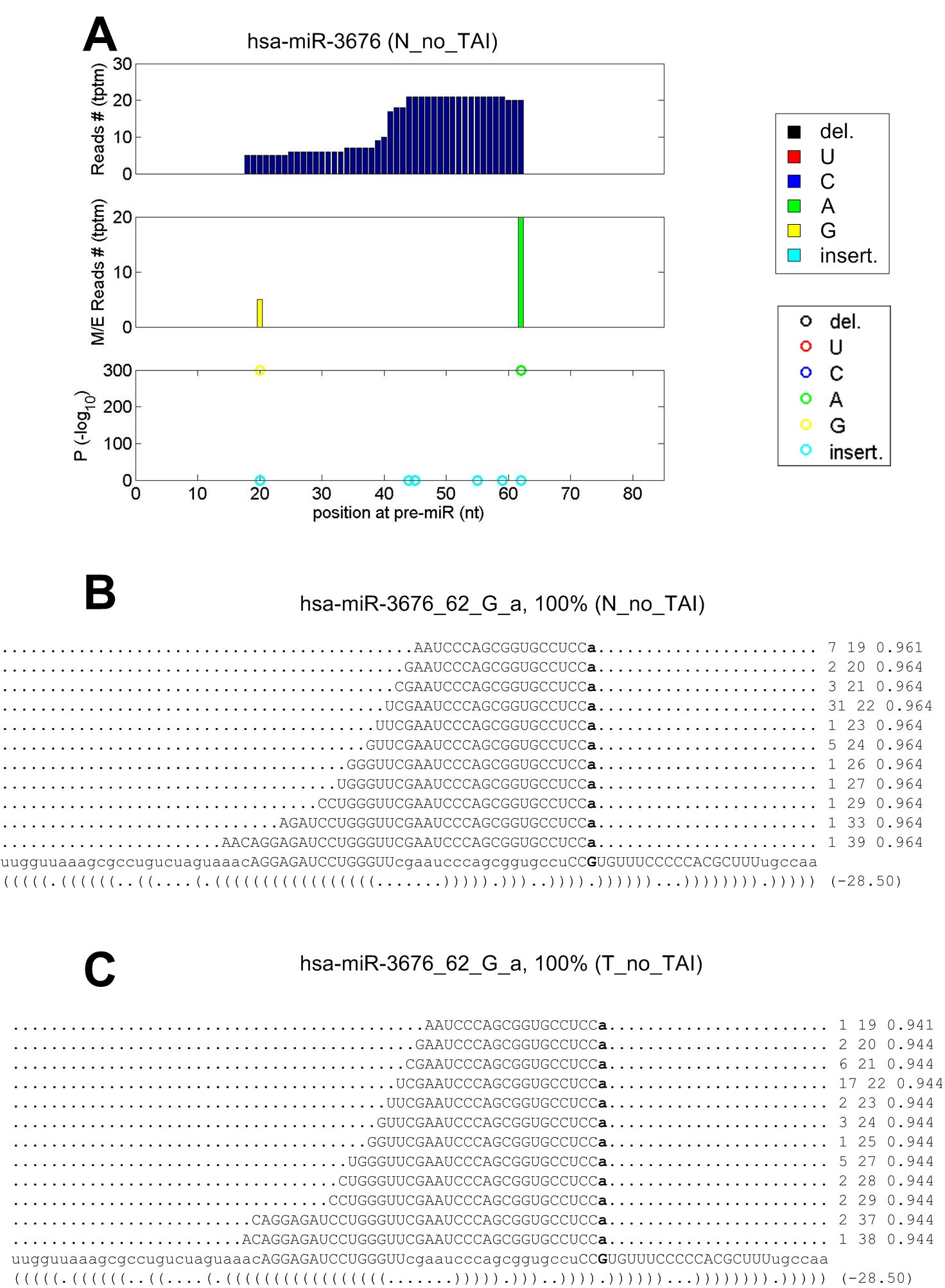

Supplement: Additional File 6 — Figure S4 -- The example of a potential SNP on hsa-miR-3676. (A) is a schematic view of hsa-miR-3676. (B) to (C) are the reads supporting hsa-miR-3676_62_G_a in the N_no_TAI and T_no_TAI data set, respectively. Legend idem to those of Figure 2. [file 1471-2164-15-S9-S11-S6.jpg]
